# Assessing the Risk Factors Associated with Malaria in the Highlands of Ethiopia: What Do We Need to Know?

**DOI:** 10.3390/tropicalmed2010004

**Published:** 2017-03-01

**Authors:** Élodie Anne Vajda, Cameron Ewart Webb

**Affiliations:** 1 School of Public Health, University of Sydney, Sydney, NSW 2006, Australia; 2 Department of Medical Entomology, NSW Health Pathology, Westmead Hospital, Westmead, NSW 2145, Australia; cameron.webb@health.nsw.gov.au; 3 Marie Bashir Institute for Infectious Diseases and Biosecurity, University of Sydney, Sydney, NSW 2006, Australia

**Keywords:** malaria, transmission, epidemic, outbreak, surveillance, climate, *Anopheles*, population mobility

## Abstract

Malaria has been Ethiopia’s predominant communicable disease for decades. Following the catastrophic malaria outbreak in 2003–2004, the Federal Ministry of Health (FMoH) took drastic public health actions to lower the burden of malaria. The FMoH achieved significant declines in malaria mortality and incidence, and recently declared its objective to achieve malaria elimination in low malaria transmission areas of Ethiopia by 2020. However, while the overall malaria prevalence has decreased, unpredictable outbreaks increasingly occur irregularly in regions previously considered “malaria-free”. Such outbreaks have disastrous consequences on populations of these regions as they have no immunity against malaria. The Amhara Region accounts for 31% of Ethiopia’s malaria burden and is targeted for malaria elimination by the FMoH. Amhara’s epidemiological surveillance system faces many challenges to detect in a timely manner the unpredictable and irregular malaria outbreaks that occur in areas of otherwise low transmission. Despite the evidence of a shift in malaria transmission patterns, Amhara’s malaria control interventions remain constrained to areas that are historically known to have stable malaria transmission. This paper discusses the influence of temperature and precipitation variability, entomological parameters, and human population mobility on malaria transmission patterns across the Amhara Region, and in particular, in areas of unstable transmission. We argue that malaria epidemiological surveillance systems can be improved by accounting for population movements in addition to environmental and entomological factors. However, to date, no study has statistically analyzed the interplay of population dynamics on environmental and entomological drivers of malaria transmission.

## 1. Introduction

Malaria continues to be the most significant mosquito-borne disease globally but it holds a particularly heavy burden across many countries in Africa where in 2015, 88% of global cases and 90% of global deaths due to malaria were recorded [1]. One country suffering a substantial burden of disease due to malaria is Ethiopia. Prior to 2005, malaria was Ethiopia’s number one communicable disease, causing 5–10 million cases and approximately 70,000 deaths per annum. Following the disastrous malaria epidemic of 2003–2004 that resulted in 3,143,163 reported cases in 2003, and in 5,706,167 cases in 2004 [2], Ethiopia distributed from 2004 to 2012, 46 million long-lasting insecticidal nets (LLINs), performed indoor residual spraying (IRS) in 70% of the targeted households, and dispensed 9 million doses of free malaria treatment in health care facilities [3,4]. A detailed account of Ethiopia’s malaria control and treatment interventions can be found in the WHO’s World Malaria Report (2008).

The FMoH generated substantial positive outcomes: malaria cases dropped from 3,759,960 cases in 2006, to 1,214,921 cases in 2007 [2]. The WHO now considers Ethiopia to be “on-track” to achieving a 50%–75% decrease in malaria case incidence [5]. Nonetheless, 75% of Ethiopia’s landmass is still considered ‘malarious’, and 68% of Ethiopia’s national population total of 84 million inhabitants are at risk of malaria [3,4,5,6,7,8,9]. Moreover, timely detection of malaria is challenged by inconsistent commitment in passive surveillance across Ethiopia, lack of resources for adequate data collection, and high staff turnover [10,11]. Surveillance must be strategically adapted to the shifting epidemiologic trend in Ethiopia, which is seeing declines in malaria transmission [12]. To sustain these declines in malaria transmission, it is critical to identify subpopulations that are at elevated risk in Ethiopia. Mobile human populations and an extensively rural setting makes access to health care facilities inconsistent, and thus, data collected by malaria in-patient surveillance system might not depict an accurate picture of the status of malaria transmission.

The spatial occurrence of malaria is largely determined by climate variables. Malaria occurs where there is sufficient precipitation to establish suitable mosquito habitats for vector species, adequate humidity to enable high activity and survival of mosquitoes, and elevated temperatures to sustain quick gonotrophic and sporogonic cycles [13,14]. Accordingly, because temperatures fall with rising elevation, temperature determines the elevation limit of stable malaria transmission. Notwithstanding the impact on parasite cycles, environmental conditions may not be suitable for mosquitoes at higher elevations. However, studies of *Anopheles* spp. elsewhere in Africa indicate that changing climate may shift the altitudinal limits on some species [15].

Malaria is considered endemic in regions of stable *Plasmodium* transmission but malaria outbreaks often also arise in regions of unstable transmission (i.e., non-endemic) (1500–2500 meters above sea level (masl)), which are characterized by climate that is marginally suitable for mosquitoes. In such regions, outbreaks of malaria might be irregular, but higher than normal rates of malaria transmission often occur, and symptoms are exacerbated due to the low immunity of human populations inhabiting these areas [16]. In addition to being immunologically naïve, these human populations may not be aware of risk associated with local mosquito populations, and this may result in reduced adoption of personal protection measures. The perception of low malaria risk has been identified as a contributing factor to non-compliance of bednets in Ethiopia [17].

Motivated by the significant decline in malaria cases and deaths over the last decade, the FMoH of Ethiopia declared its objective for the year 2020 to achieve elimination of malaria within targeted geographical areas that historically have low malaria transmission [4]. While the overall prevalence of malaria in Ethiopia is decreasing, previously considered ‘malaria-free’ (i.e., above 2000 masl) zones, such as the Ethiopian highlands, are increasingly reporting epidemic malaria transmission and face irregular and unpredictable malaria outbreaks [6].

## 2. Malaria in the Amhara Region, Ethiopia

The Amhara Region is one of the nine geographic divisions in Ethiopia and is located in the north-west of the country. Covering approximately 150,000 km^2^, it is Ethiopia’s second most populated state, and comprises a number of districts (*woredas*, Ethiopia’s third-level administrative units) in which the initial malaria elimination program is planned. Amhara’s drastic altitudinal range from 506 m at the Blue Nile Gorge, to 4533 m at Ras Dashen, makes the prediction of the timing and geographic range of malaria outbreaks particularly challenging. In 2012, the Amhara Region counted 1,127,241 malaria cases, out of a population of 19,867,817 inhabitants [4]. However, as malaria intervention efforts in Ethiopia are concentrated in areas below 2000 masl, intervention activities in highland regions are minor and compromise the overall malaria control strategies [18].

The major malaria vector in Ethiopia is *Anopheles arabiensis*, while *An. pharaoensis*, *An. funestus*, and *An. nili* remain secondary vectors [19]. Additional vectors include *An. chrysti*, *An. cinereus*, and *An. demeilloni* [19]. Both *An. arabiensis* and *An. pharaoensis* have been found breeding in regions above 2100 masl, and recent malaria reports from the highlands suggest that these vectors breed up to 3000 masl [20]. The predominant *Plasmodium* species is *P. falciparum*, accounting for 60% of malaria cases across Ethiopia, while *P. vivax* accounts for 40% of malaria cases across the country [21]. Generally, during increased malaria transmission and epidemics, *P. falciparum* prevails over *P. vivax*, whereas during low malaria transmission periods, *P. vivax* predominates [22]. However, since 2011, *P. falciparum* is on the decline, whereas *P. vivax* is proportionally on the rise. This may result from Ethiopia’s neglect of the ‘less fatal’ *P. vivax* parasite to heavily focus on the public health threat posed by the *P. falciparum* parasite [21]. Additionally, a recent study shows that a higher prevalence of *P. falciparum* occurs in the lowlands, whereas as a higher prevalence of *P. vivax* occurs in the highlands [22]. Other contributing reasons to these recent shifts might be spatiotemporal climate variability and the development of *P. vivax*’s chloroquine resistance [22,23].

To address the increasing risk of epidemic malaria in the Amhara Region, there needs to be a better understanding of malaria transmission drivers in highland regions [4,6,14,18,21,24,25,26,27]. For instance, Alonso et al. (2011) [26] demonstrated that a temperature increase attributable to climate change directly caused the recent rise in malaria incidence in the East African Highlands. Similarly, Minakawa et al. (2002) [28] proposed that a changing climate might be contributing to changed distributions of malaria vectors in Kenya. There is, however, some debate internationally as to the role of changing temperatures and malaria risk in highland regions [25].

The role of human population and human activity further complicate the identification of risk factors driving malaria. Alemu et al. (2014) [18] emphasize the significance of mobile populations traveling between high- and low-transmission regions. As people move between endemic malaria regions in which both the vectors and parasites occur, there is a risk that both might be introduced into non-endemic regions. Furthermore, human activity itself, through deforestation, water management and urbanization might also influence malaria risk [29,30]. Thus, understanding how the interactions between environmental and climatic variables, entomological parameters, and human population dynamics influence malaria outbreak risk is essential to sustain adequate and effective malaria control in Ethiopia.

In this light, this paper discusses risk factors associated with malaria in the Amhara Region with specific focus on the influence of (1) temperature and precipitation variability; (2) entomological parameters that contribute to malaria outbreaks in non-endemic areas; and (3) the current evidence for the impact of population movements on malaria outbreaks. Finally, this paper concludes with knowledge gaps and remarks on the implications of the dynamics of such drivers on malaria transmission for achieving the FMoH’s objective to eliminate malaria transmission in historically “low-transmission” areas of Amhara Region by 2020.

## 3. Key Factors Driving Malaria Risk in Amhara Region

### 3.1. Climate Variables

The major malaria transmission in Ethiopia occurs from September to December, subsequent to the predominant rainy season (June–September) [6]. The Ethiopian highlands witness two malaria transmission seasons annually: the major one follows the June to September rainy season from September to December, and the moderate transmission period follows the February to March rainy season from April to May [21,31]. Malaria epidemics in regions of unstable transmission occur in 5- to 8-years cycles, although in recent years, these have reduced to 1- to 2-year cycles [6,21,31]. The most recent malaria epidemic in the Amhara Region took place from 2003 to 2005. Since that period, malaria outbreaks have been smaller and highly localized [6,14,32].

Malaria epidemics in non-endemic regions of the Amhara Region largely occur as a result of above-average temperatures and precipitation [33,34]. Precipitation is linked to the prolonged occurrence of water bodies for female mosquitoes to lay their eggs and for successful larval development. These egg-laying sites are most common in deforested regions, such as agricultural fields. Studies in Ethiopia have identified a diverse range of immature mosquito habitats [35] including ephemeral habitats that become more productive during periods of above-average rainfall. However, excessive precipitation can also wash away mosquito larvae and thus drastically reduce mosquito population density [6].

Temperature impacts the length of larval development, mosquito survival, and parasite development. Elevated temperatures accelerate the development rate of both the mosquito larvae and of the *Plasmodium* parasites [24]. With increased development rates, a higher number of mosquito generations arise, and in greater abundance. As temperatures increase, malaria transmission rates increase up to a threshold level of approximately 37 °C that is rarely surpassed [36]. The increased malaria transmission rates with higher temperatures result from elevated feeding rates of the female adult mosquito, thus increasing the likelihood of malaria transmission to humans. In addition, the length of the sporogonic cycle (parasite development inside the mosquito) decreases as temperature increases beyond optimal temperatures [34].

Elements that increase ambient temperatures in the Amhara Region include land clearance for agricultural purposes, and the El Niño-Southern Oscillation event (ENSO) which occurs every 2 to 10 years [14,37]. In the Ethiopian highlands, El Niño events cause higher than normal rainfall, along with elevated temperatures, thus creating ideal conditions for malaria vectors [38]. However, an El Niño event is not systematically followed by malaria epidemics in the Ethiopian highlands, which blurs the causal relationship of El Niño with epidemic malaria in the highlands [38].

The effects of increased rainfall on malaria transmission and risks for malaria outbreaks are compounded by increased temperatures and increased humidity [39]. However, several studies have found that elevated temperatures in the absence of humidity, reduces mosquito longevity, and hence, their capacity to spread *Plasmodium* [38]. Whether increased temperatures or increased rainfall is the main driver of malaria epidemics in the highlands remains unclear, and to date, various studies find differing results [14,39,40,41]. It is plausible that a combination of interactions between environmental factors influence the entomological and parasitology drivers of increased transmission.

Previous studies demonstrate the occurrence of time lags ranging from weeks to months between climate anomalies and elevated malaria transmission rates [6,32,33,42]. These time lags following a climatic anomaly are due to the time needed for mosquitoes to reach the adult stage, to then acquire and transmit the malaria parasite, and finally, for malaria symptoms to manifest in the human host [33].

Climate change may expand the distribution of malaria to the highlands through epidemics or through the gradual expansion of the parasite’s territory in response to warming, and perhaps as temperatures gradually increase above normal along the altitudinal gradient. While a changing climate will influence the variables that influence malaria transmission (i.e., temperature, precipitation), the change to altitudinal distribution of malaria in Amhara and other highland regions of Ethiopia and sub-Saharan Africa is still largely unclear [43]. In their study, Siraj et al. [43] strictly examined the effect of interannual changes in temperature on a spatiotemporal scale in the highlands of both Colombia and Ethiopia. They found that in the warmer than normal years, the altitudinal distribution of malaria increased, therefore suggesting that climate change may increase the altitudinal distribution of malaria. However, *Plasmodium* transmission is not driven by a single variable such as temperature. In fact, malaria is a disease whose transmission pattern is varyingly driven by a range of factors such as access to health care, lifestyle, vector behavior, deployed vector control measures, and human behavior and migration [25,43]. Thus, the effect of climate change on malaria may likely be minor in comparison to the potential impact of public health interventions and better socioeconomic conditions [44].

### 3.2. Entomological Parameters

A diverse range of malaria vector mosquitoes is found in Ethiopia, as mentioned above, but the main vector is *An. arabiensis*, a member of the *An. gambiae* species complex containing at least seven species, all of which transmit malaria parasites. The main secondary vectors are *An. pharaoensis*, *An. funestus*, and *An. nili* [19]. Entomological monitoring in the Amhara Region is incomplete.

Behavioral and biological variations across mosquito species, such as habitat associations and associated climatic and environmental drivers of population abundance, host-seeking behavior, and resistance to insecticides, influence the nature of malaria outbreaks [38,45]. Thus, consideration must be given to vector behavior when evaluating the role of mosquitoes in the epidemiology of malaria [46]. For example, because *An. funestus* breeds in permanent water bodies, its population remains stable, and thus, *An. funestus* rarely causes malaria outbreaks [38]. However, because *An. funestus* is a highly efficient malaria vector, it plays an important part in the increase of malaria endemicity, and consequently, it may contribute to triggering malaria outbreaks [38,47]. Other adult mosquito species show reduced flight behavior, and can therefore cause clustered malaria outbreaks, rather than widespread ones [38]. Moreover, mosquitoes are highly adaptable to environmental change, and have behavioral changes in response to antimalarial interventions [45].

The predominant malaria vectors occurring in the highlands are considered endophilic, i.e., they feed and rest indoors [48]. Social norms such as the Ethiopian tradition of cooking, sleeping, and tethering their livestock inside their homes increases the indoor temperature, thus creating a suitable environment (enhanced temperature and relative humidity) for mosquitoes. Therefore, house interiors are where mosquitoes generally bite and transmit malaria [48]. As a consequence, dwelling design and construction can influence the exposure of individuals to mosquitoes. Many homes in the Ethiopian highlands are inadequately constructed; open eaves and doors that do not properly fit in their door frames are both common problems, and allow mosquitoes to come inside. Thus, high indoor vector densities are associated with malaria transmission [48].

In studies conducted by Peterson et al. (2009) [49] and Alemu et al. (2013) [50], malaria transmission in the Ethiopian highlands was found to be highly clustered. Homes that were either poorly constructed or situated near a breeding site had much higher malaria transmission rates, and vector density, than groups of houses better built and/or farther away from breeding sites. Peterson et al. (2009) [49] reinforces the revealed association between the malaria vectors’ endophilic behavior and malaria transmission rates within highland communities. Alemu et al. (2013) [50] emphasizes the heterogeneity of malaria transmission in small-scale geographical areas.

However, recent studies find that vectors such as *An. arabiensis* and *An. pharoensis* are not exclusively endophilic/-phagic, with a behavioral shift observed in feeding time from later evenings when humans are indoors, to earlier evenings when humans are still outdoors [30,51,52]. Prior to the deployment of IRS as the main vector control strategy in Ethiopia in the 1960s, *Anopheles* were recorded to feed mostly indoors [30]. Following such widespread vector control measures, these vectors are increasingly reported to be feeding and resting outdoors [51]. In fact, studies spanning the last decade, find that peak feeding hours for *An. arabiensis* are in the early evening when people are still active outdoors [30,35]. Consideration should be given to a potential shift to outdoor host-seeking behavior in response to indoor vector control measures [45]. This suggests that vectors of malaria are capable of future behavioral (and perhaps physiological) changes to malaria control interventions. Consequently, it should be considered that the simultaneous use of multiple and diverse vector control strategies (e.g., larval control, topical control, removal of egg-laying sites) are necessary to mitigate widespread vector behavioral changes in response to the broad use of a single vector control strategy, such as LLINs.

### 3.3. Human Population Dynamics

Recent studies demonstrate that human migration and short-term travel are risk factors for malaria transmission in the Amhara Region as well as for other regions of Ethiopia [8,10,18]. A study published in 1991 by Nega and Meskal [53] found evidence suggesting that high rates of malaria transmission in the 1980s were due to large population movements from non-malarious highlands to lowland malarious regions. Such large-scale movements stemmed from drought and famine in the highlands, attracting desperate highlanders, without immunity to malaria, to the fertile lowlands where risk of exposure was high. Deressa et al. (2006) [54] highlighted the dramatic rise in malaria transmission rates in the 1990s following resettlement programs as malaria-infected populations returned to their homes in the highlands. The concept of population movement, whether mass migrations, or smaller, sub-populations, influencing malaria transmission rates is not new [55,56,57,58]. However, there is very little data on the influence of highlanders travelling back and forth between the highlands and the lowlands on both malaria transmission rates in the highlands and on *Plasmodium* transport back to the highlands [8,55].

Large-scale population movements in Ethiopia are largely due to seasonal agricultural work opportunities [54]. The lowlands of the Amhara Region are very fertile and thrive on extensive farming of cash crops such as sorghum and sesame. Since both farming and malaria transmission are dependent upon precipitation, the agricultural season (June to October) occurs simultaneously with the rainy season (July to September), and thus, overlaps with the malaria transmission season (September to December). As a result, highland farm workers, having travelled to the lowlands for farm work, are especially prone to malaria infection due to low immunity [18,55].

In their study on migrant farm workers in the North Gondar Zone of the Amhara Region, Schicker et al. (2015) [8] found that migrant workers tend to live in temporary housing unfit for protection against mosquitoes (e.g., absence of bednets and poorly constructed walls and roofs). Similarly, male workers often slept outdoors at night during peak transmission periods, increasing exposure to mosquitoes [21]. Notwithstanding exposure to malaria vectors under these circumstances, there may not be an inherent awareness of malaria risk, predisposing these individuals to greater risk, given the lack of personal protection measures.

As well as human behavior exposing individuals to malaria risk, movement between regions may also be a risk factor. Schicker et al. (2015) [8] observed that migrant workers in the lowlands of North Gondar zone mostly originate from the highlands and highland fringes of Amhara and exhibit a higher prevalence of malaria than usual estimates of malaria transmission in the lowlands. These temporary farm workers return home at the end of the agricultural season, and this return to the highlands facilitates *Plasmodium* transmission in areas of Amhara that would otherwise have limited malaria transmission [22].

Several studies have found that malaria prevalence is particularly high amongst mobile young male adults (15 years+) [8,18,59]. Alemu et al. (2014) [18] explains that highland females are at lower risk than highland males because females are more likely to stay in the highlands and more inclined to make use of LLINs. Highland males are also at greater risk because they travel more frequently to the lowlands for agricultural work, job seeking, and social events [10,18]. In their survey conducted in the Dabat District of the Amhara Region, Alemu et al. (2014) [18] found that half of highland malaria-infected patients in their study had not travelled to lowland malarious regions, thus suggesting some degree of highland village malaria transmission.

Moreover, Kibret et al. (2010) [30] noted substantial heterogeneity in malaria transmission between Ziway villages, despite being separated from each other by only a small distance. This heterogeneity resulted from a difference in irrigation practices for agricultural activities and, subsequently, suitable habitats for vector mosquitoes. This finding may indicate that small-scale population movements are a risk factor for malaria transmission in the highlands [30]. This risk factor is compounded by the heterogeneity of Amhara’s topography, vector parameters, and by human-driven landscape changes, such as irrigation systems, dams, and deforested land for pastoral and agricultural activities [10,30].

Thus, although there have been increased efforts to control malaria transmission in Amhara, small and mobile sub-population groups that are difficult to track, might be at higher risk of malaria infection and may delay its elimination from certain areas, or may even re-introduce malaria in areas where it had been eliminated [10]. Moreover, even though Alemu et al. (2014) [18] and Yukich et al. (2013) [10] found that men travelled more than women for agricultural purposes, Yukich et al. (2013) [10] observed higher malaria incidence in females than in males, whereas Alemu et al. (2014) [18] reported the reverse, thereby demonstrating the complexity of the malaria epidemiology. A better understanding of the populations’ demographics and their likely exposure to malaria is critical in maintaining low transmission rates.

Finally, the current and persistent population growth across Ethiopia [60], in combination with the impacts of climate change on Ethiopia’s natural resources, farming practices, and climate variables, will likely cause more mass migrations between lowlands and highlands [59]. Rapid population growth further compounds this by challenging the FMoH’s malaria control interventions as demand for health care and disease prevention rapidly increases. In settings that are resource-constrained, this may have a snowball effect, such that malaria transmission increases as authorities struggle to keep up with the demand for health care and malaria prevention, and as the environment remains highly stressed by climate change and unsustainable land use practices that favor malaria transmission.

## 4. Knowledge Gaps and the Questions They Pose

One of the critical issues to be addressed by local authorities is the gap in current understanding of how climate variability may influence the altitudinal distribution of malaria vectors together with incident rates of malaria locally. Broadly, the distribution of malaria vectors in Africa is predicted to shift in response to climate variability [61]. With increasing quality and availability of remote sensing data, it might be possible to predict altitudinal movement of key vector species such as *An. arabiensis* [62]. There will remain uncertainty about local-scale altitudinal shifts in temperature and rainfall and their resulting impact on malaria risks.

Notwithstanding the influence that these environmental changes may have on the presence and abundance of suitable vector species, there will also be epidemiological and socioeconomic factors influencing transmission rates. A better understanding of the relationships between climate variables, vector populations, and malaria transmission risk is necessary so that predictive models [63] that assist public health resource allocation to reduce risk can be developed. However, understanding how short-term changes in spatial rainfall distribution drive local vector populations is required.

It is likely that climate change will have varying effects on malaria and its vectors across the latitudinal gradient in different regions of the world. Drought may increase malaria incidence because water bodies are reduced to small pools which *Anopheles* species prefer for egg-laying [64]. This might be compounded by the fact that in rural settings, drought is associated with famine, which weakens the immune system and make the famished population significantly more vulnerable to malaria. Indeed, in Ethiopia, periods of famine due to drought have been followed by elevated malaria transmission [54].

Therefore, as one of the suspected effects of climate change is altered rainfall patterns (as already observed in Ethiopia) [65], and thus increased frequency of drought, it can be argued that if Ethiopia is successful in mitigating the impact of drought on its population health and therefore reducing the population movement between regions of unstable and stable malaria transmission, then malaria incidence may not see a drastic rise following drought. In this light, the impact of climate change on the expansion of malaria transmission may be influenced, not only by how mosquito populations respond to changed environmental conditions, but by how humans respond to these changes as well.

Beyond the role of climate variability on malaria transmission rates, other factors will influence future risk associated with regions of unstable malaria. Chloroquine resistance in *P. falciparum* and to a lesser degree, *P. vivax*, has often been designated as the key culprit for facilitating the expansion of malaria to previously “malaria-free” regions [66]. In Ethiopia in 1996, resistance to chloroquine in both *P. falciparum* and *P. vivax* was reported, and in 1998, sulphadoxine-pyrimethamine (SP) replaced the chloroquine regimen to treat uncomplicated infections of *P. falciparum* and mixed *P. falciparum* and *P. vivax* [67]. However, *P. falciparum* quickly developed resistance against SP, and in 2004, artemether-lumefantrine (AL) replaced SP to treat uncomplicated infections of *P. falciparum* and mixed *P. falciparum* and *P. vivax* [67]. On the other hand, chloroquine prevails as the first-line treatment for *P. vivax* infections in Ethiopia (including other sub-Saharan countries).

More recently, Mekonnen et al. (2014) [67] found that chloroquine-sensitive *P. falciparum* parasites had returned in Ethiopia. This is likely due to the period of withdrawal of chloroquine. Moreover, Beyene et al. (2016) [68] also found that in Ethiopia, *P. vivax* was highly successfully treated with chloroquine. These are encouraging results because chloroquine is cheap, and easy to prepare and use. Thus, as resistance to drugs is recurrent, using combination therapies that include short-acting drugs with various pharmacodynamic properties should be explored as a means to reduce drug resistance [67]. However, as noted by Beyene et al. (2016) [68], additional studies are needed to verify these results because there remain widespread reports of failure to treat *P. vivax* with chloroquine across Ethiopia.

As previously discussed, a recent study demonstrates that a higher prevalence of *P. falciparum* occurs in the lowlands, whereas *P. vivax* has a higher prevalence in the highlands [22]. This is an important finding to further examine because if this trend is true, it might be useful to deploy different treatment combinations in the highlands and lowlands, depending on how *P. vivax* and *P. falciparum* are found to respond to these drug therapies. Concurrently, monitoring the population movement between the lowlands and the highlands is relevant to better track the dynamics of these two *Plasmodium* species.

Consideration should also be given not only to changes in resistance of the parasites to anti-malarial medications, but also in vector resistance to insecticides. An understanding of the likely genetic basis of specific behavioral resistance traits, and how surveillance programs should be implemented to best monitor changes in these traits is important. This will be especially relevant to insecticide resistance in malaria vectors [69] which has already been reported [70,71]. However, the actual impact of insecticide resistance on malaria transmission rate has yet to be fully understood [72]. Approaches are being developed to assist the mapping of insecticide resistance in *Anopheles* spp. in Africa [73] and thus, ensuring sufficient data be collected from region of unstable malaria transmission will greatly contribute to the development of strategic control initiatives. Where resistance develops, the sustainability of integrated malaria management will be threatened and adaptive strategies are required.

## 5. Concluding Remarks

In 2015, the FMoH reiterated its determination to make considerable strides towards achieving malaria elimination by 2020 in historically “low transmission” areas of Ethiopia, including several districts of the Amhara Region. However, this declaration is not novel. In fact, the FMoH had also targeted 2015 for the same objective [4]. Under the USAID President’s Malaria Initiative, the Malaria Operational Plan of fiscal year 2015 for Ethiopia noted the unpredictability of malaria outbreaks in zones of unstable malaria as a major hindrance to achieving malaria elimination in low transmission areas [74]. However, the most recent Malaria Operational Plan (fiscal year 2017) is focused on endemic regions of malaria, thus neglecting regions of unstable malaria transmission [75].

If Ethiopia is to achieve its goal of eliminating malaria transmission in low transmission areas of the Amhara Region by 2020, then Ethiopia must scale up the coverage of their malaria interventions in regions of unstable transmission. While achieving this may face many operational barriers, it is critical as the movement of highlanders to the lowlands may compromise elimination efforts by (1) reintroducing *Plasmodium* to regions where malaria control efforts have achieved substantial decreases in malaria prevalence; and (2) triggering malaria outbreaks in areas of unstable transmission as travelling malaria-infected highlanders return to their homes in the highlands.

Scaling-up malaria interventions (e.g., distribution of LLINs) in regions of unstable transmission is challenging because malaria control measures are not constantly necessary throughout the year, since transmission is inconsistent. Such control measures are often costly, and require reliable coordination and cooperation between public health actors to ensure highland populations receive their LLINs in a timely manner to secure optimal protection against malaria during outbreaks. Additionally, the sole distribution of LLINs may not be sufficient as the vectors are gradually shifting to feeding during the day rather than during the night. Thus, finding an appropriate control measure that can be easily and quickly deployed at lower costs is challenging. Community-based larval control is cheap and has proven effective in reducing mosquito population density and in reducing malaria because it is a localized disease [76]. However, this type of control measure is most effective when used in combination with other measures, and its long-term sustainability has yet to be tested. Therefore, other control measures should further focus on increasing the awareness of highlanders of personal protective measures, such as the use of topical repellents, and eliminating egg-laying sites around homes.

To the best of our knowledge, no study has yet simultaneously statistically analyzed environmental factors, entomological parameters, and human population dynamics in relation to malaria transmission. Such a study is necessary to improve our understanding of malaria transmission dynamics and to improve Ethiopia’s epidemiologic malaria surveillance programs.
